# Therapeutic modulation of neurogenesis to improve hippocampal plasticity and cognition in aging and Alzheimer's disease

**DOI:** 10.1016/j.neurot.2025.e00580

**Published:** 2025-04-02

**Authors:** Mostafa Mostafa, Ahmed Disouky, Orly Lazarov

**Affiliations:** Department of Anatomy and Cell Biology, University of Illinois at Chicago, Chicago, IL, 60612, USA

**Keywords:** Hippocampus, Neurogenesis, Aging, Alzheimer's disease, Therapy

## Abstract

Alzheimer's disease is characterized by progressive memory loss and cognitive decline. The hippocampal formation is the most vulnerable brain area in Alzheimer's disease. Neurons in layer II of the entorhinal cortex and the CA1 region of the hippocampus are lost at early stages of the disease. A unique feature of the hippocampus is the formation of new neurons that incorporate in the dentate gyrus of the hippocampus. New neurons form synapses with neurons in layer II of the entorhinal cortex and with the CA3 region. Immature and new neurons are characterized by high level of plasticity. They play important roles in learning and memory. Hippocampal neurogenesis is impaired early in mouse models of Alzheimer's disease and in human patients. In fact, neurogenesis is compromised in mild cognitive impairment (MCI), suggesting that rescuing neurogenesis may restore hippocampal plasticity and attenuate neuronal vulnerability and memory loss. This review will discuss the current understanding of therapies that target neurogenesis or modulate it, for the treatment of Alzheimer's disease.

## Hippocampal Neurogenesis in the Adult Brain

Postnatally, new neurons are formed in a discrete niche in the subgranular zone (SGZ) of the dentate gyrus in the hippocampus [1]. The formation of these new neurons can be traced back to self-renewing and multipotent adult neural stem cells (NSCs) that are located in the SGZ of the dentate gyrus (DG) [[2], [3], [4]]. In the rodent, NSCs were classified into two distinct categories based on their distinctive shapes, ability to multiply, and the presence of specific molecular proxies [5]. Type 1 ​cells have radial processes that extend over the entire granule cell layer and branches out in the inner molecular layer of the DG. Typically, they are distinguished by distinct co-expression of molecular proxies such as GFAP, Sox2 and Nestin. These cells are thought to be quiescent and give rise to actively self-amplifying, non-radial type 2 ​cells. Intermediate progenitor cells, which express Sox2 and Nestin but not GFAP, give rise to neuroblasts. Surviving neuroblasts extend dendrites to the outer molecular layer of the DG to receive input from the entorhinal cortex (EC). They send axonal projections to the CA3 subfield of the hippocampus to connect with hilar interneurons, mossy cells, and CA3 pyramidal cells, that differentiate into glutamatergic dentate granule cells and become part of the existing neuronal circuitry. Interneurons play a crucial function in multiple aspects. GABA-induced depolarization and excitation regulates the dendritic development of newborn neurons in the adult brain [6]. Initially, the formation of neurons is influenced by ambient GABA [6,7]. Levels of ambient GABA are regulated by interneuron activity. The integration of new neurons into the preexisting functional circuitry starts from tonic GABA activation to GABAergic synaptic innervation, followed by glutamatergic synaptic innervation [6]. Tonic GABA activation provides an initial depolarization of these new neurons and constitutes the majority of GABA-induced activation during the initial integration process [6]. As the process progresses, the balance between inhibition and excitation becomes crucial in determining the preference of new neurons to respond to incoming stimuli, hence directing activity toward these new neurons [8]. Immature neurons exhibit distinct electrophysiological characteristics compared to their mature counterparts. This is characterized by higher amplitude and lower induction threshold for long-term potentiation (LTP), manifested by increased synaptic plasticity [9].

The process of neurogenesis and integration of new neurons encompasses several essential stages. Multiple tiers of regulation, consisting of both inherent and external processes, have been discovered at every stage. For instance, morphogens such as Notch, sonic hedgehog (Shh), Wingless (Wnts), and bone morphogenetic proteins (BMPs), which play crucial roles in early development of the nervous system, act as signals that regulate the maintenance of hippocampal NSCs, their activation, and fate determination. Growth factors (e.g., EGF, bFGF) and cell-cycle signals play a role in the proliferation of activated NSCs ad NPCs. Neurotrophins (e.g., BDNF, NGF), cytokines (e.g., IGF-1, ILs), hormones, neurotransmitters, endogenous elements such as miRNAs, transcription factors and epigenetic factors underlie regulation of neurogenesis. An extensive description of these factors is reviewed elsewhere [10]. Some of these factors are produced endogenously, locally by other residents of the niche, or transported from either periphery via the BBB or from innervating neurons. The latter includes input from a variety of neuronal types and brain regions, including GABAergic local interneurons, acetylcholinergic of the septum, dopaminergic of the ventral tegmentum, serotonergic of the raphe nuclei.

Similar to other stem cell niches in the body, the SGZ neurogenic niche harbors a milieu that supports an equilibrium between the maintenance of NSCs and the facilitation of their differentiation. In addition to the neurogenic lineage, the niche consists of glia, endothelia, pericytes, immune cells, neuronal processes and extracellular matrix. The niche creates a distinct permissive environment that is composed of specialized extracellular matrix, chemical signals, and cell – cell contact and communication. Neurogenesis is critically dependent on the interaction with the vasculature, referred to as the "vascular niche" [11]. An intricate connection was described between endothelial cells, astrocytes and hippocampal NSCs [12,13]. Vascular dependency was further evident by the presence of end-feet of nestin-positive type I NSCs on the vasculature in the SGZ [14]. Vascular factors, such as vascular endothelial growth factor (VEGF), are essential or necessary for neurogenesis [15]. In addition, peripheral signals cross the blood brain barrier via interaction with the vasculature.

Transcription factors (TFs) play a pivotal in regulating neurogenesis, i.e., self-renewal, proliferation, fate determination and differentiation of NSCs into either neuronal or glial lineages [[16], [17], [18], [19]]. For a comprehensive review of TF in adult neurogenesis see Beckervordersandforth et al., 2015 [18]. Here, we provide a few examples of several of these key TF. Glioma-Associated Oncogene Homolog 1 (Gli1), a member of the Kruppel family of zinc finger proteins, is activated by the sonic hedgehog signal transduction cascade and regulates NSC self-renewal and proliferation in the adult DG [20]. The activity and nuclear localization of Gli1 is negatively regulated by p53 in an inhibitory loop [21]. Likewise, deletion of forkhead box protein O3 (FoxO3) in the SVZ and SGZ resulted in decreased self-renewal, leading to the depletion of the NSC pool [22]. In addition, RE1 silencing transcription factor (REST) expression in NSCs maintains NSCs in a quiescent state [23]. Kruppel-like factor 9 (Klf9) was reported to regulate symmetric cell division of NSCs [24]. In addition, it is implicated in neuronal maturation [25], which may suggest that the role of some TF is stage-specific. SRY-related high-mobility-group box 2 (Sox2) is expressed in both radial and horizontal NSCs and is pivotal in NSC self-renewal [16]. Its expression decreases in postmitotic type 3 neuroblasts as they mature into neurons [26]. Conditional deletion of Sox2 resulted in the depletion of type 1 and type 2 NSCs in the adult SGZ, accompanied by a decrease in granule neurons [27]. Notably, the interaction between Sox2 and the long non-coding RNA, RMST, is essential for cell fate determination in NSCs [28]. Furthermore, Sox2 represses the expression of the neurogenic differentiation 1 (NeuroD1), a basic transcription factor required for the survival and maturation of new neurons (Gao et al., 2009). Relief of Sox2-dependent repression of NeuroD1 by Wnt signaling is necessary for neurogenesis to progress [29]. In the aged brains, reduced expression of wild-type p53-induced protein 1 (WIP1) leads to increased inhibition of Wnt signaling via Dickkopf 3 (DKK3), ultimately contributing to decreased neurogenesis. Hence, DKK3 may be a therapeutic target for age-linked reduced neurogenesis [30].

Canonical Notch signaling, mediated through the recombination signal binding protein for immunoglobulin kappa J (RBPJκ) pathway, is crucial for promoting and maintaining Sox2 expression in NSCs [31]. Notch/RBPJκ signaling also promotes the expression of the bHLH gene Hes5 in quiescent and actively dividing early progenitors in the hippocampus [32]. Paired box gene 6 (Pax6) sustains the multipotent state of early progenitors [33]. Pax6 heterozygous rats exhibited reduced NSC proliferation and new neuron generation [32,33].

The orphan nuclear hormone receptor tailless (TLX) is required for NSC self-renewal in the SGZ and SVZ [34,35]. It interacts with HDAC3 and HDAC5 to repress the expression of cell cycle regulatory factor p21 and tumor suppressors such as pten and p53, promoting NSC proliferation [36,37]. Whether FoxO3, TLX, and REST act in concert or as distinct pathways in regulating adult neurogenesis is unclear. TLX activates Wnt signaling, promoting NSC self-renewal in the presence of growth factors [35]. However, in their absence, Wnt signaling may enhance NSC differentiation through the activation of NeuroD1 [29,35]. The specific outcome of Wnt signaling likely depends on stimuli and neurogenic niche context to maintain homeostasis of neurogenesis. Additional TF networks determine fate specification and differentiation of new neurons. Achate-schute complex homolog-like 1 (Ascl1), in combination with other TFs, is essential for NSC fate specification. Manipulating Ascl1 levels and contextual expression alters progenitor fate. Ectopic Ascl1 expression in the hippocampus generates oligodendrocytes at the expense of granule neurons [38]. T-box brain gene 2 (Tbr2) expression in intermediate progenitors facilitates neuronal lineage progression [39] and cyclic AMP response element-binding protein (CREB) is implicated in neuronal maturation and plasticity [40]. Overall, TFs coordinate a complex sequence of events regulating neurogenesis in a spatial and temporal – specific way. This should be considered when designing their manipulation as a therapeutic approach for the amelioration of age-related reduced neurogenesis and cognitive decline. The molecular mechanisms and signaling pathways involved in regulating neurogenesis are summarized in (Table 1).

**Table 1:** Molecular mechanisms and signaling pathways regulating neurogenesi.

Molecular Mechanism	Signaling Pathway/Molecule	Cell Type	Experimental Model	Results in Neurogenesis	References
Notch signaling	Notch → RBPJκ → Hes5, Sox2	Neural stem Cells (NSCs)	Mouse SVZ & Hippocampus	Maintains NSC quiescence and promotes Sox2 expression in NSCs	[16,31,32]
Sonic Hedgehog (Shh) signaling	Shh → Ptch1 → Smo → Gli1	NSCs & Neural Progenitor Cells (NPCs)	Mouse embryonic brain, in vitro cultures	Regulates NSC self-renewal and proliferation; negatively regulated by p53	[20]
Wnt/β-catenin signaling	Wnt → Frizzled/LRP5/6 → β-catenin → NeuroD1, DKK3	NSCs, NPCs, newborn neurons	Mouse hippocampus, in vitro NSC cultures	Regulates NSC self-renewal and differentiation; NeuroD1 required for neuronal survival	[29,30,35]
Bone Morphogenetic Proteins (BMPs)	BMPs → BMPR1 → Smad1/5/8	NSCs	Mouse SVZ, adult hippocampus	Regulates maintenance and activation of NSCs	[16,105]
Fibroblast Growth Factors (FGFs)	bFGF → FGFR	NPCs, radial glial cells	Mouse embryonic cortex	Promotes proliferation of activated NSCs and NPCs	[10,294]
Epidermal Growth Factor (EGF)	EGF → EGFR	NSCs, NPCs	In vitro human NSCs, rodent models	Enhances proliferation and survival of NSCs	[10,295]
Neurotrophins	BDNF, NGF, NT-3 → TrkB	Newborn neurons, NPCs	Rodent hippocampus, in vitro NSCs	Supports neuronal survival, differentiation, and synaptic integration	[296]
Insulin-like Growth Factors (IGFs)	IGF-1 → IGF-1R	NSCs, NPCs, neurons	Mouse dentate gyrus, in vitro NSCs	Enhances neurogenesis and promotes neuronal survival	[297]
Interleukins & Cytokines	IL-6, IL-1β, TNF-α	NSCs, NPCs	Inflammatory rodent models	Modulate neurogenesis, inflammation, and cellular communication	[298]
GABAergic signaling	GABA → GABA_A/GABA_B receptors	Newborn neurons	Hippocampal cultures, in vitro models	Initial depolarization of new neurons, synaptic integration	[[6], [7], [8]]
Glutamatergic signaling	Glutamate → AMPA, NMDA receptors	Newborn neurons, mature neurons	Mouse hippocampus	Excitatory synaptic integration in new neurons	[[299], [300], [301]]
Transcription Factors (TFs)	Gli1, FoxO3, REST, Klf9, Sox2, NeuroD1, TLX, Ascl1, Tbr2, CREB, RBPJκ, Pax6	NSCs, NPCs	In vitro cell cultures, mouse brain	Regulates self-renewal, proliferation, and differentiation of NSCs	[[20], [21], [22], [23], [24],[26], [27], [28],[33], [34], [35]]
Epigenetic Regulators	HDAC3, HDAC5, p53, PTEN, RMST (lncRNA), DNA methylation	NSCs, NPCs	Mouse brain, in vitro cultures	Controls NSC gene expression, differentiation, and cell cycle regulation	[21,36,37]
Vascular Niche	VEGF, endothelial cells, pericytes, CXCL12	NSCs, endothelial cells	Rodent models, human brain	Provides support for NSC proliferation and neurogenesis	[302,303]
Peripheral signals & systemic Factors	Hormones (cortisol, estrogen), BBB interaction	NSCs, NPCs	Rodent models, human samples	Modulates neurogenesis by integrating external physiological signals	[301,304]

**NSCs**: Neural Stem Cells, ​**NPCs**: Neural Progenitor Cells, ​**BDNF**: Brain-Derived Neurotrophic Factor, ​**NGF**: Nerve Growth Factor, ​**NT-3**: Neurotrophin-3, ​**TrkB**: Tropomyosin Receptor Kinase B, ​**IGF-1**: Insulin-like Growth Factor 1, ​**IGF-1R**: Insulin-like Growth Factor 1 Receptor, ​**IL-6**: Interleukin-6, ​**IL-1β**: Interleukin-1 Beta, ​**TNF-α**: Tumor Necrosis Factor Alpha, ​**GABA**: Gamma-Aminobutyric Acid, ​**AMPA**: α-Amino-3-Hydroxy-5-Methyl-4-Isoxazolepropionic Acid, ​**NMDA**: N-Methyl-d-Aspartate, ​**VEGF**: Vascular Endothelial Growth Factor, ​**CXCL12**: C-X-C Motif Chemokine Ligand 12, ​**BBB**: Blood-Brain Barrier, ​**HDAC**: Histone Deacetylase, ​**lncRNA**: Long Non-Coding RNA, ​**RBPJκ**: Recombination Signal Binding Protein for Immunoglobulin Kappa J Region, ​**Hes5**: Hairy and Enhancer of Split-5, ​**Sox2**: SRY-Box Transcription Factor 2, ​**Shh**: Sonic Hedgehog, ​**Ptch1**: Patched 1, ​**Smo**: Smoothened, ​**Gli1**: Glioma-Associated Oncogene Homolog 1, ​**Wnt**: Wingless/Integrated, ​**LRP5/6**: Low-Density Lipoprotein Receptor-Related Protein 5/6, ​**NeuroD1**: Neuronal Differentiation 1, ​**DKK3**: Dickkopf-Related Protein 3, ​**BMPs**: Bone Morphogenetic Proteins, ​**BMPR1**: Bone Morphogenetic Protein Receptor Type 1, ​**Smad1/5/8**: Mothers Against Decapentaplegic Homolog 1/5/8, ​**bFGF**: Basic Fibroblast Growth Factor, ​**FGFR**: Fibroblast Growth Factor Receptor, ​**EGF**: Epidermal Growth Factor, ​**EGFR**: Epidermal Growth Factor Receptor, ​**CREB**: cAMP Response Element-Binding Protein, ​**FoxO3**: Forkhead Box O3, ​**REST**: RE1-Silencing Transcription Factor, ​**Klf9**: Kruppel-Like Factor 9, ​**TLX**: Nuclear Receptor Subfamily 2 Group E Member 1 (also known as NR2E1), ​**Ascl1**: Achaete-Scute Family BHLH Transcription Factor 1, ​**Tbr2**: T-Box Brain Protein 2, ​**Pax6**: Paired Box 6, ​**HDAC3**: Histone Deacetylase 3, ​**HDAC5**: Histone Deacetylase 5, ​**p53**: Tumor Protein p53, ​**PTEN**: Phosphatase and Tensin Homolog, ​**RMST**: Rhabdomyosarcoma 2 Associated Transcript (lncRNA), ​**DNA methylation**: DNA Methylation.

## Neurogenesis in the olfactory bulb

In rodents, another neurogenic niche exists in the subventricular zone, where a substantial number of migrating neuroblasts are generated from neural stem cells (NSCs) and travel through the rostral migratory stream (RMS) to reach the olfactory bulb (OB). There, they differentiate into interneurons [[41], [42], [43]]. Current evidence suggests that the human RMS contains very few migrating neuroblasts post 2 years of age, which appear as singlets or in pairs rather than forming chains [[44], [45], [46]]. It is estimated that less than 1 ​% of neurons are replaced in the human OB over a span of 100 years [47]. Cross-species comparative analysis revealed that an RMS exists in different forms in rodents, primates and humans [[48], [49], [50]]. Likewise, the duration of neuronal maturation varies and is longer in primates compared to rodents [[51], [52], [53]].

Notably, in the neonatal rodent brain, a small proportion of neuroblasts derived from the V-SVZ migrate also to the neocortex, a process that persists for a few weeks after birth [54]. Similarly, in neonatal and juvenile marmosets, neuroblasts with a monopolar or bipolar morphology were observed in the neocortex [51]. However, the survival rate and maturation potential of these juvenile-born neuroblasts are limited [51]. In the infant human brain, chains of neuroblasts have been observed migrating radially to the frontal lobe of the neocortex [55]. Furthermore, a novel migratory pathway in the human brain, termed the medial migratory stream (MMS), branches from the proximal limb of the RMS and extends to the ventromedial prefrontal cortex [45]. These migratory routes for human neuroblasts appear to remain active for at least a few months after birth before ceasing. Additionally, the presence of neuroblasts has been reported in other brain regions, including the striatum of both marmosets [51] and humans [56,57].

Similar to the hippocampus, the density of neuroblasts and immature neurons decline with age in the SVZ-OB. [58,59]. While the functional significance in the SVZ-olfactory bulb of rodents was well characterized [60,61], the functional significance of neuroblast migration to other brain regions is yet to be fully elucidated. Here, we focus mainly on hippocampal neurogenesis due to its implications for learning and memory, cognitive function and dysfunction in AD.

## Hippocampal Neurogenesis, Cognitive decline and Alzheimer's disease

Immature and new neurons in the adult DG play important roles in different forms of learning and memory that depend on the hippocampus. Numerous studies manipulated levels of hippocampal neurogenesis in rodent models and showed an effect of learning and memory. Among others, these studies utilized irradiation, chemical or genetic manipulation to target neurogenesis. Manipulations that led to reduced or diminished levels of neurogenesis resulted in impaired performance in various cognitive tasks, including contextual discrimination (pattern separation), spatial navigation, long-term spatial memory retention, spatial pattern discrimination, trace conditioning, contextual fear conditioning, clearance of hippocampal memory traces, and reorganization of memory to extra-hippocampal substrates. On the other hand, manipulations that led to the enhancement of neurogenesis, such as environmental enrichment, running, deep brain stimulation, or genetic manipulation, led to improved performance in these tasks (for comprehensive review see Refs. [1,2]. Mounting evidence has linked impaired neurogenesis to cognitive deterioration in Alzheimer's disease (AD). AD is manifested by progressive memory loss and cognitive impairment, leading to dementia. The accumulation of β-amyloid (Aβ) deposits and neurofibrillary tangles precede memory loss and starts in the hippocampal formation, particularly layer II of the entorhinal cortex (ECXII) [62]. New neurons in the DG send their axons into the outer molecular layer of the dentate gyrus to form synapses with neurons of ECXII. The latter are highly vulnerable and are one of the first neurons to die in AD [63]. The former have been shown to be impaired in mouse models of AD for a comprehensive review see Ref. [64] and in human patients [[65], [66], [67]]. Hippocampal neurogenesis was shown to play a causative role in learning and memory deficits in AD. For example, Hollands et al. reported that depletion of neurogenesis by crossing APPswe/PS1ΔE9 mice with nestin-driven thymidine kinase (δ-HSV-TK) mice exacerbated learning and memory deficits of the APPswe/PS1ΔE9 mice [68]. Conversely, Mishra et al. crossed 5XFAD mice with NestinCreER^T2^; Bax^fl/fl^ mice to conditionally increase the survival of newly born neurons, and hence neurogenesis, and showed that enhancing neurogenesis in FAD mice restored learning and memory and the recruitment of immature neurons to memory circuits. Immature neurons recruited to memory circuits had restored dendritic spine density and gene profile. Inactivating immature neurons after this enhancement reversed the memory benefits. Key AD-related genes like App, ApoE, and Adam10 were notably affected, indicating that impaired neurogenesis contributes to memory loss in Alzheimer's disease [69,70]. Hippocampal neural stem cells (NSCs) derived from postmortem tissue of AD patients showed decreased proliferation and/or viability and enhanced senescence compared to NSC isolated from aging with no cognitive impairments [71]. Synaptic degeneration in the outer molecular layer was observed in AD human patients [72]. In addition to ECXII, neuronal loss takes place in the CA1 region in the hippocampal formation, and gradually in other cortical areas [73]. Several pathological factors have been implicated in deficits in neurogenesis in AD, offering insights into the molecular underpinnings of this dysfunction. The Accumulation of extracellular Aβ, a hallmark of AD, was shown to contribute to impairments in the neurogenic niche in AD [[74], [75], [76]]. Elevated dose of APP or its amyloidogenic metabolites were shown to compromise neurogenesis and exacerbate cognitive decline [[77], [78], [79]]. Tau pathology including the accumulation of neurofibrillary tangles disrupt neurogenic regulators, such as Musashi1, and impairs neuronal proliferation and differentiation, suggesting that tau-associated disruptions in cytoskeletal dynamics and intracellular signaling pathways may directly impair hippocampal neurogenesis (for a comprehensive review see Ref. [80]. In addition, Mitochondrial dysfunction and oxidative stress may be contributors to AD pathology. Among other mechanisms, mitochondrial dysfunction was proposed to mediate its effects on hippocampal neurogenesis via NeuroD1 [81]. Oxidative stress was also implicated in deficits in neurogenesis in AD. Lastly, several studies reported loss of inhibitory neurons in the cortex and hippocampus in AD [82,83]. Given the co-dependency of neurogenesis and inhibitory neurons in the DG, this could be another factor that leads to compromised neurogenesis in AD or to overall vulnerability in the DG.

### Impact of AD on adult neurogenesis in humans

In a pioneering study, Eriksson and colleagues examined postmortem brain sections of patients treated with BrdU (5-bromo-2′-deoxyuridine), a thymidine analog that incorporates into the DNA of proliferating cells. New neurons were found in the hippocampus only in the granular and subgranular zone of the DG [84]. Two years later, Roy et al. isolated NPCs from the hippocampus of adult humans and successfully maintained them in culture conditions. These NPCs were able to differentiate into mature granular neurons, providing further evidence for active neurogenesis in this region [85]. However, this method did not allow accurate quantification of the number of new neurons in vivo. In 2013, Spalding et al. provided quantitative evidence for the existence of hippocampal neurogenesis in the adult human [86]. Using ^14^C birth dating by quantifying the isotope concentration in the genomic DNA of neurons, Spalding et al. estimated the rate of new neurons in the human dentate gyrus as 700/day. Moreover, they determined that 35 % of the DG neurons undergo turnover with a renewal rate of 1.75 % per year. In light of that, they concluded that a full renewal of the neuronal population of the dentate gyrus takes place in the course of a lifetime [86].

Immunohistochemical analysis of postmortem brain tissue yielded a series of controversial results. Dennis et al. investigated the extent of neurogenesis in 23 postmortem hippocampal samples from human subjects aged 1–59 years. The authors were not able to detect hippocampal neurogenesis in the adult human dentate gyrus. Moreover, they reported that the only proliferating cellular population that they were able to detect in the DG or its adjacent parenchyma were microglia [87]. Sorrells et al. investigated the extent of neurogenesis over the lifespan of humans using 19 pre- and postnatal samples of brain tissue [88]. The authors reported that while the pre- and postnatal dentate gyrus up to a year old contained a great number of immature neurons, they were not able to detect any immature neurons in the DG of and suggested that hippocampal neurogenesis comes to a halt during adulthood [88]. In contrast, three recent reports have shown evidence for the persistence of hippocampal neurogenesis during adulthood. Using similar proxy markers, Boldrini et al. investigated postmortem hippocampal samples obtained from 28 individuals aged 14–79 years and detect thousands of NPCs and immature neurons in the DG of adult humans [89]. Another study investigated the difference in AHN in 45 cognitively impaired patients between the ages of 52 and 97, distributed across the six neuropathological Braak stages of the disease. The number of DCX + cells detected was consistently higher in healthy individuals of any age than in AD patients, regardless of age [90]. Tobin et al. investigated the extent of AHN in postmortem hippocampal tissue of 18 individuals aged 79–99 years and categorized them into three categories based on their cognitive performance: healthy aging, MCI, and AD [65]. In addition to an evidence for the existence of AHN up to the 10th decade of life in all 3 cognitive diagnoses, they reported that the number of neuroblasts was lower in MCI and AD patients and correlated with their cognitive diagnosis, thus indicating that AHN is impaired early in the AD disease course and has the potential of being a therapeutic target of early intervention [65]. Moreno-Jiménez et al. investigated AHN in postmortem tissue obtained from 13 healthy aging individuals aged 43–87 years and from 45 AD patients aged 43–87 years [66]. They provided evidence for the existence of AHN until 90 years of age, while seeing a moderate reduction in AHN during aging. Moreover, using stereological counting, the authors reported that AHN declines drastically in AD patients and is correlated with the patients’ Braak stage [66]. Taken together, the latter studies provided evidence that the extent of neurogenesis is associated with cognitive status, while the specific mechanisms remain to be elucidated. This controversy could have several reasons, including differences in experimental conditions, such as antigen retrieval, the quality of the postmortem tissue, pre-recorded information on patients, which may shed light on co-morbidities and their brain pathophysiology, method of cell quantification, variability between human subjects [66]. For instance, the majority of the tissue used by Sorrells et al. came from patients with epilepsy known to have diminished neurogenesis [91]. Thus, using standardized protocols and cohorts may help alleviate the controversy.

### Sequencing-based profiling of hippocampal neurogenesis

Considering the intricate cellular composition of the neurogenic niche in humans, single-cell transcriptomics has emerged as a promising technique to advance our understanding of this niche [[92], [93], [94], [95]]. Recent studies employing single-cell RNA sequencing (scRNA-seq) in the DG of mice have demonstrated that the adult neurogenic niche functions as a spatially defined, complex multicellular system [96,97]. Within this localized area, neural stem cells (NSCs) and their progeny establish a tightly regulated continuum of cellular states [19,96], supported by various niche-resident cell types, including diverse neuronal populations, niche-specific microglia, astrocytes, oligodendrocytes, and specialized vasculature [96,98,99]. scRNA-seq has also been utilized to profile the human hippocampus [67,94,[100], [101], [102], [103]]. Three recent comprehensive studies focusing on the adult human DG have yielded conflicting outcomes: two reported the presence of immature neurons [67,101], while one study failed to identify any neurogenic populations [100]. Zhou et al. also examined the presence of neurogenesis in a cohort of AD patients and reported marked reduction in the number of immature neurons in AD compared to aging with no cognitive deficits [67]. This controversy highlights an urgent need for further investigation that comprehensively characterizes the neurogenic niche in the human hippocampus and elucidates its alterations in AD patients. Expanding these studies with diverse cohorts, including well-defined AD patient groups, and integrating advanced techniques like epigenetics, spatial transcriptomics and proteomics is critical for the resolution of these conflicting results for offering a deeper understanding of the molecular impairments occurring within the neurogenic niche in AD, which is instrumental to pave the way for targeted therapeutic interventions.

## Modulation of neurogenesis

### Neurotrophic growth factors mediate adult neurogenesis

The identification of signals that are altered in the aging or diseased DG may be possible therapeutic targets or open up new avenues in that regard. For example, imbalance between bone morphogenetic proteins (BMP2 and BMP4) and noggin, where BMP is upregulated is implicated in reduced neurogenesis in depression and aging [[104], [105], [106]]. One of the homeostatic mechanisms affected in the aged brain that might contribute to the decline in neurogenesis is the Wnt signaling pathway. A downregulation of Wnt ligands and an upregulation of Wnt inhibitors (Dkk1 or sFRP3) has been observed in the aged brain that impairs neurogenesis [107,108] and the Wnt β-catenin signaling has been proposed as a potential therapeutic target**.** Endogenous neurotrophic growth factors, such as nerve growth factor (NGF), brain-derived neurotrophic factor (BDNF), glia-derived neurotrophic factor (GDNF), and insulin-like growth factor 1 (IGF-1), play vital roles in promoting development, proliferation, and differentiation of NSCs in the central nervous system [109,110]. Many of these neurotrophic factors activate tropomyosin-related kinase (Trk) receptors, including type A and B, initiating intracellular signaling cascades that govern NSC self-renewal and fate determination [111]. The indispensability of BDNF-TrkB signaling in the enhancement of hippocampal neurogenesis and the survival of newly generated neurons during adult neurogenesis has been unveiled [112]. Considering the significant contributions of neurotrophic factors to neuronal plasticity and function, alterations in levels and expression of their respective receptors are associated with a multitude of psychiatric and neurodegenerative disorders [113,114]. For example, diminished NGF levels and impaired NGF-TrkA signaling in forebrain cholinergic neurons have been reported in both AD patients and aged rats [115,116]. BDNF and NGF are implicated in supporting neurogenesis in the adult brain [117,118]. Therefore, enhancing these factors in neurodegenerative conditions such as AD holds promise for therapeutic intervention. A study conducted on adult rats revealed that intracerebroventricular (ICV) infusion of BDNF not only stimulated the production of new neurons but also enhanced their survival in the RMS and the OB [119]. Similarly, the continuous infusion of NGF through ICV in young adult rats facilitated adult hippocampal neurogenesis and the survival of new neurons [117]. Notably, ICV infusion of NGF or BDNF resulted in enhanced cognitive functions in rats by promoting neurogenesis in the hippocampus [118,120]. Another study on adult rats indicated that peripheral infusion of IGF-1 led to increased proliferation of progenitor cells and selectively induced hippocampal neurogenesis [110]. One caveat is their relatively large size and polar molecular structure. To overcome the impermeability of the BBB led to the direct infusion of purified neurotrophic factors into the brain through ICV injection. A clinical trial involving AD patients who received ICV infusion of NGF reported an enhancement in cognitive function [121]. Despite the slight cognitive improvements observed in AD patients with NGF infusion in the brain, long-term administration through ICV was linked to severe side effects such as hyper-innervation of cerebral blood vessels [122], hypophagia [123] and neuropathic pain [121]. Collectively, these studies suggest that the invasive delivery of neurotrophic factors may be constrained by side effects, potentially limiting their clinical advantages. Additionally, achieving clinically beneficial concentrations of neurotrophic factors through delivery has proven to be arduous.

Subsequent studies have endeavored to devise methods that enable the transport of adequate levels of neurotrophic factors to the brain. One strategy involves implanting cells that express neurotrophic factors into affected brain regions. A phase I clinical trial involving the injection of NGF-expressing fibroblasts into the nucleus basalis of AD patients demonstrated sustained expression of NGF for up to one-year post-injection, accompanied by cognitive enhancement [124]. The precise mechanisms underlying the cognitive improvement remained unknown; however, an autopsy conducted on a single individual five weeks after implantation revealed robust growth responses of cholinergic neurons [124]. While this study did not explore neurogenesis or specifically target engraftment of NGF-expressing cells to adult neurogenic regions [124], it did showcase prolonged expression of neurotrophic factors compared to infusion, without any enduring adverse effects [124]. One drawback of this approach is the requirement for numerous invasive injections directly into the brain, posing a significant risk of neural injury and subcortical hemorrhage [124]. It is yet to be determined whether similar approaches could be employed to specifically enhance adult neurogenesis in future trials.

Short peptide mimetics are viewed as a promising therapeutic strategy due to their ability to target neurotrophic receptors and elicit similar signaling effects compared to their full-length counterparts [125]. Furthermore, these peptide mimetics exhibit high specificity, potentially enhancing receptor activation by targeting either TrKA or TrkB receptors, while also improving bioavailability and reducing proteolysis [125]. Investigations into the effectiveness of these peptide mimetics have been conducted in various animal models. For example, a small peptide mimetic of BDNF, known as Peptide B-5, was found to stimulate the expression of BDNF, and its receptor TrkB in primary hippocampal neuronal cultures, suggesting an enhanced neurotrophic impact [126]. Similarly, small peptide mimetics of CNTF, a compound with established neurotrophic properties [127], have been created and assessed for their therapeutic advantages [128]. The subcutaneous administration of CNTF small peptide mimetics, such as peptide 6, was shown to enhance adult neurogenesis in the dentate gyrus, improve neuronal plasticity, and boost spatial memory in mice [128]. Moreover, the incorporation of adamantylated glycine groups into CNTF small peptides, resulting in a pentamer named Peptide 021, significantly enhanced the permeability of the blood-brain barrier [129]. When administered peripherally, Peptide 021 stimulated neurogenesis and neuronal maturation in the hippocampus of adult mice [129]. Furthermore, the increase in neurogenesis observed after Peptide 021 treatment was linked to the enhancement of learning and memory [129]. In a separate study, oral administration of Peptide 021 in aged rats reversed the age-related decline in new neurons in the hippocampus [130]. Additionally, Peptide 021 was found to upregulate the expression of BDNF and TrkB receptors in both the hippocampus and cortex [130], thereby promoting BDNF-dependent adult neurogenesis [130]. Research conducted in animal models has underscored the potential therapeutic value of neurotrophic mimetics in boosting adult neurogenesis, thanks to their specificity, bioavailability, and limited adverse effects. Nevertheless, further clinical investigations into the efficacy of neurotrophic peptide mimetics are necessary to uncover their therapeutic advantages.

### Small molecules

Small molecules that can modulate specific neurogenic signals or processes may have a therapeutic potential. For example, protein kinases of the protein kinase C (PKC)- activating diterpene small molecule has been shown to facilitate NSC proliferation in neurogenic niches when injected intracerebroventricularly. PKC stimulates the release of growth factors that stimulate NSC proliferation [131]. In another study, Gomez-Oliva et al. (2023) demonstrated that treating SAMP8 mice, a model of accelerated aging, with a 12-deoxyphorbol diterpene from 4 to 6 months of age prevents cognitive decline and enhances neurogenesis. Normally, these mice, which show AD - like symptoms and cognitive deficits by 6 months, experience disrupted neurogenesis as they age. However, the 12-deoxyphorbol diterpene treatment resulted in the production of fully mature, correctly positioned neurons in the hippocampus, effectively counteracting the aging process and preventing cognitive impairment [132].

ACEA, harmine, D2AAK1, methyl 3,4-dihydroxybenzoate, and shikonin may induce neuronal proliferation/differentiation through the activation of pathways: MAPK ERK, PI3K/AKT, NFkB, Wnt, BDNF, and NPAS3. Combinations of these compounds can potentially transform somatic cells into neurons. This transformation process involves the activation of neuron specific transcription factors such as NEUROD1, NGN2, ASCL1, and SOX2, which subsequently leads to the transcription of downstream genes, culminating in the transformation of somatic cells into neurons [133].

The maturation of human pluripotent stem cell (hPSC)-derived neurons mimics the protracted timing of human brain development, extending over months to years for reaching adult-like function. Prolonged in vitro maturation presents a major challenge to stem cell-based applications in modeling and treating neurological disease. Therefore, scientists designed a high-content imaging assay based on morphological and functional readouts in hPSC-derived cortical neurons which identified multiple compounds that drive neuronal maturation including inhibitors of lysine-specific demethylase 1 and disruptor of telomerase-like 1 and activators of calcium-dependent transcription. A cocktail of four factors, GSK2879552, EPZ-5676, N-methyl-d-aspartate and Bay K 8644, collectively termed GENtoniK, triggered maturation across all parameters tested, including synaptic density, electrophysiology and transcriptomics. Maturation effects were further validated in cortical organoids, spinal motoneurons and non-neural lineages including melanocytes and pancreatic β-cells [134].

### Synthetic compounds that modulate AD pathology and their effect on neurogenesis

We and others have shown that key signals in AD regulate adult hippocampal neurogenesis. For example, γ-secretase/PS1 regulate neuronal differentiation of NPCs in the adult hippocampus [135], and soluble APPα facilitates proliferation of hippocampal NPCs [77]. Likewise, tau metabolism is critical for neuronal development, differentiation and function. For comprehensive review of AD signals and hippocampal neurogenesis see Lazarov and Marr [136,137]. Thus, it would be reasonable to assume that drugs targeting pathological hallmarks of AD may affect neurogenesis and support its function. Below we summarize the current knowledge in this regard.

### Amyloid-targeting medication

Amyloid immunotherapy refers to antibodies that target amyloidosis at different stages. In addition to amyloid clearance, studies in FAD mouse models showed improved performance in learning and memory. Treatment of APP/PS1 mice with anti-Aβ showed enhanced survival of new neurons in the dentate gyrus. These neurons exhibited increased dendritic branching and spine densities [138]. FDA-approved anti-amyloid monoclonal antibody infusion include Donanemab, Lecanemab, Aducanumab. Aducanumab is a human IgG1 monoclonal antibody that targets oligomer and fibril forms of beta-amyloid that was discontinued in 2024. Among its effects were reduction of amyloid pathology and improve impaired cognition [[139], [140], [141], [142]]. Treatment of 5XFAD mice with low dose of Aducanumab in combination with enhanced delivery via focused ultrasound led to increased number of new neurons and CREB signaling [143].

**Tarenflurbil**, is a member of the non-steroidal anti inflammatory drugs (NSAID) a selective β-amyloid-lowering agent, modulates γ-secretase activity, reducing β-amyloid aggregations [144].

**Gantenerumab**, a monoclonal antibody targeting aggregated Aβ in the brain, exhibits sustained binding to cerebral Aβ and effectively reduces small Aβ plaques by eliciting microglia-mediated clearance, without disturbing systemic Aβ clearance [145]. Combination treatment with gantenerumab and RO5508887, a BACE inhibitor, demonstrated enhanced anti-amyloid effects, suggesting a potential clinical relevance of combination therapies in AD [146].

**scyllo-inositol and neotrofin** - combining Aβ-targeting **scyllo-inositol** with neotrofin, a purine hypoxanthine derivative, which increases neurotrophins, enhanced neuronal survival and differentiation, proposing a combination therapy targeting Aβ-pathology and neurotrophin deficits as a potential treatment for AD [147].

### Acetylcholinesterase inhibitors

**Donepezil**, a second-generation cholinesterase inhibitor, has been shown to enhance phosphorylation of cAMP response element-binding protein (CREB), crucial for the survival and function of new neurons in the DG [148] (Table 2). Additionally, donepezil improves hippocampal neurogenesis in a rat model of vascular dementia, a common type of AD, by upregulating BDNF expression [149]. Furthermore, cognitive enhancement via increased neurogenesis in the DG has been associated with donepezil administration [148,150].

**Table 2:** Synthetic drugs that modulate neurogenesis.

Synthetic Drug	Model	Mechanisms	Findings	Ref
Donepezil	Rat/vascular dementia	↑ AChEI ↑ MAPK signaling	Enhanced hippocampal neurogenesis Improved learning and memory	[273]
	Normal rats	↑ AChEI ↑ CREB signaling ↑ BDNF	Enhanced hippocampal neurogenesis Improved survival of newborn neurons	[274]
	Young and aged mice	↑ AChEI	Enhanced proliferation of neural stem cells (NSCs)	[275]
Galantamine	Normal mice	↑ AChEI ↑ Activation of M1 muscarinic receptor ↑ Activation of α7 nicotinic receptor ↑ Hippocampal IGF2 expression	Enhanced hippocampal neurogenesis Improved survival of newborn neurons Increased proliferation of neural progenitor cells	[151]
	In vitro (cortical cultures); in vivo (CD1 mice)	↑ AChEI	Enhanced hippocampal neurogenesis	[277]
	Old rats	↑ AChEI ↑ Activation of M1 muscarinic receptor ↑ spatial memory ↑ Activation of α7 nicotinic receptor	Enhanced hippocampal synaptic plasticity	[278]
Rivastigmine	Olfactory bulbectomized (OBX) mice	↑ MAPK signaling ↑ Protein kinase B (Akt) ↑ 5-HT1A receptor stimulation	Enhanced hippocampal neurogenesis Improved survival and proliferation of neural progenitor cells	[279]
	In vitro (cortical cultures)	↑ BuChEI ↑ AChEI	Improved synaptic function Increased cell viability	[280]
	In vitro (PC12 ​cells and PHB cultures); in vivo (APP/PS1 mice)	↑ Non-amyloidogenic pathway ↑ α-secretase activity (ADAM-9, -10, and −17, as well as sAPPα.)	Increased cell viability Decreased β-amyloid peptide in the hippocampus	[281]
Methylthioninium chloride	In vitro (primary neuronal cultures and organotypic slice cultures); in vivo (JNPL3 mice)	↑ Autophagic signaling	Reduced phospho-tau levels Induced autophagy Increased tau clearance	[282]
	NMRI mice	↑ Brain cytochrome c-oxidase activity	Improved spatial learning and memory Enhanced anti-tau activity	[283]
	Mild AD patients	↑ Inhibition of tau aggregation	Reduced clinical and brain atrophy Improved clinical outcomes	[284]
Saracatinib	AD transgenic mice	↑ Fyn kinase inhibitor	Improved spatial learning and memory Increased synapse density Reduced microglial inflammation and tau pathology	[285]
Masitinib	Patients with mild-to moderate AD	↑ Fyn kinase inhibitor	Reduced cognitive deficits	[286]
Valproic acid	1321N1 Astrocytoma cells	↑ S100B suppression	Increased astroprotective effects Improved glial cell viability	[287]
	APP/PS1 mice	↑ GSK3beta inhibition ↑ Wnt/β-catenin signaling	Improved spatial learning and memory Reduced AβP deposition Increased synaptic density Enhanced neuroprotective effects	[288]
	APP/PS1 mice	↑ GSK3beta inhibition ↓ NF-κB signaling pathway	Reduced number of apoptotic cells Improved spatial learning and memory Reduced AβP deposition Decreased activation of astrocytes and microglia Increased neurite outgrowth	[289]
Lithium	People with amnestic mild cognitive impairment	↑ GSK3beta inhibition	Reduced CSF phospho-tau concentration Improved cognitive performance	[290]
	Aged TgCRND8 mice	↑ GSK3beta inhibition ↑ Wnt/β-catenin signaling	Enhanced cell proliferation and survival Improved hippocampal neurogenesis Increased cognitive functions Reduced β-amyloid production	[291]
	Tgtau30 mice	↑ GSK3beta inhibition	Reduced tau hyperphosphorylation Decreased neurofibrillary tangle density	[292]
Tarenfurbil	Patients with mild AD	γ-secretase enzyme ↓ Amyloidosis	Reduced amyloidosis, no improvement in cognitive activities	[293]
Bapineuzumab	Patients with mild-to moderate AD	↓ Amyloidosis	Decreased CSF phospho-tau concentration, no improvement in cognitive activities	[294]
	Patients with mild-to moderate AD	Anti-β-amyloid Antibody ↓ Amyloidosis	Reduced amyloidosis Decreased β-amyloid accumulation Reduced CSF phospho-tau concentration	[295]
Gantenerumab	APP London mice	Anti-β-amyloid Antibody ↓ Amyloidosis	Reduced amyloidosis Decreased AβP formation	[296]
	PS2APP mice	Anti-β-amyloid Antibody ↓ Amyloidosis	Reduced amyloidosis Decreased β-amyloid plaque deposition	[297]
Scyllo-inositol/neotrofin	TgCRND8 mice	↑ Induction of neurotrophins	Increased neuronal survival and differentiation Reduced β-amyloid peptide Enhanced hippocampal neurogenesis	[298]
Epothilone D	PS19 tau mice	↑Microtubule-stabilizing agent	Increased density and axonal integrity Reduced memory and cognitive deficits	[299]
	PS19 tau mice	↑Microtubule-stabilizing agent	Enhanced hippocampal neuroprotective activity Reduced forebrain tau pathology Improved cognitive performance	[300]
Davunetide (NAP)	Patients with mild cognitive Impairment	↑Microtubule-stabilizing agent	Improved cognitive function	[301]
	DM-Tau-transgenic mice	↑Microtubule-stabilizing agent	Increased neuroprotection activity Improved spatial learning and memory	[302]
Memantine	Adult mouse	↑BDNF ↑ NMDA receptor stimulation	Enhanced cell proliferation and survival Increased hippocampal neurogenesis	[176]
	3xTg-AD mice	↑ NMDA receptor stimulation	Reduced β-amyloid peptide and hyperphosphorylated tau Improved cognitive performance	[304]

AD Alzheimer's disease, AChEI acetylcholinesterase inhibitor, NMDA N-methyl-d-aspartate, NSCs neural stem cells, AHN Adult hippocampal neurogenesis, BDNF brain-derived neurotrophic factor, NPCs neural precursor cells, CREB cAMP response element-binding protein, GSK3beta glycogen synthase kinase 3 beta, MAPK Mitogen-activated protein kinase, IGF2 Insulin-Like Growth Factor 2, BuChEI Butyrylcholinesterase inhibitor, APP/PS1 amyloid precursor protein/presenilin-1, Akt protein kinase B.

**Galantamine**, another member of the acetylcholinesterase inhibitors (AChEIs) family, exerts positive effects on AD by enhancing the function of nicotinic and muscarinic acetylcholine receptors [151]. Chronic treatment with galantamine increased bromodeoxyuridine labeling (BrdU) uptake in cortical cultures [152]. Beneficial effects on hippocampal neurogenesis in adult mice have also been demonstrated through M1 muscarinic and α7 nicotinic receptor activation by galantamine [151]. Furthermore, galantamine enhances dopamine levels in the prefrontal cortex and promotes cell proliferation, survival, and neuronal maturation in the hippocampus in mice and rats [[151], [152], [153]].

**Rivastigmine**, a second-generation AChEI, promotes neurogenesis in the DG through stimulation of serotonin receptor type 1A (5-HT1A) [154]. Additionally, rivastigmine treatment enhances phosphorylation of protein kinase B (Akt) and extracellular signal-regulated kinase (ERK), and increases neurogenesis [154]. Importantly, rivastigmine modulates levels of α-secretase activity, directing APP processing toward the non-amyloidogenic pathway [155,156].

### Tau-targeted compounds

For a comprehensive review of tau-targeted compounds see Ref. [157]. Given the imperative role of tau in neurogenesis, it is reasonable to hypothesize that many of the tau-targeted compounds may benefit neurogenesis, however, more studies are warranted to establish this.

**Methylthioninium chloride (MTC)** inhibits tau protein aggregation and improves neuronal impairment related to tau protein [158]. Additionally, combination therapy with MTC and cholinesterase inhibitors may enhance therapeutic efficacy in AD [159]. MTC reduced tau levels through the induction of autophagy [160,161]. Combination therapy with rivastigmine and MTC showed promise in improving neuronal metabolism and reducing tau levels in AD models [159,160].

**Saracatinib and masitinib**, both tyrosine kinase inhibitors, exhibit potential in preventing tau pathology and cognitive impairments in the brain in taupathies including AD, [162]. AZD0530, a Fyn inhibitor, prevents aberrant signaling pathways associated with AD pathology [163]. Similarly, masitinib, a selective oral tyrosine kinase inhibitor, when administered as an add-on therapy to standard care, has been associated with a slower cognitive decline in AD patients [164].

**Epothilone D (EpoD)**, a microtubule-stabilizing agent, facilitated microtubule density and axonal integrity in PS19 tau transgenic mice, reduced learning and meory deficits and tau pathology. Robles-Gómez Á et al. (2023) studied Epo-D in rTg4510 mice with Aβ, P-tau, or both, assessing memory and CA1 neuron properties. Aβ and P-tau mildly impaired memory but had opposite effects on neuron excitability, with their combination worsening cognitive flexibility. Epo-D prevented most deficits but had condition-dependent side effects, highlighting the need for further research [[165], [166], [167]].

**NAP**, an octapeptide derived from activity-dependent neuroprotective protein (ADNP), attenuates Aβ-induced impairments in spatial memory and synaptic plasticity, offering promise as a therapeutic candidate for neurodegenerative diseases like AD [168]. These findings demonstrate that NAP, probably by enhancing PI3K/AKT pathway, plays an important positive role in attenuating Aβ1-42-induced impairments in spatial memory and synaptic plasticity [169].

**17β-estradiol, BDNF, and melatonin** are well-known regulators of hippocampal neurogenesis. They enhance cognitive function in AD, in part by stimulating neurogenesis in the hippocampus For a comprehensive review please see Ref. [170], [171], [172], [173], [174]. Research efforts are underway to identify pharmacological agents that target repair mechanisms, improving the proliferation of NPCs in the SGZ following AD. It has been reported that second-generation cholinesterase inhibitors like donepezil, rivastigmine, and galantamine are versatile pharmaceuticals that can modulate hippocampal neurogenesis by activating the cholinergic system [175]. Various pharmacological substances with potential to increase neurogenesis and ameliorate cognitive function in AD therapy are described below. Targeting cortisol.

**Memantine**, a NMDA receptor antagonist, that was reported to improve stem cell proliferation and neuronal differentiation in the adult mouse hippocampus by reducing β-amyloid aggregation [176]. Treatment with memantine restores cognition, reduces levels of Aβ and tau pathology, and prevents Aβ-induced synaptic dysfunction [177]. Zisiadis et al., (2023) demonstrated that a high, non-clinical dose of memantine (50 mg/kg) increased cell proliferation in the intact brain by 72 % and reduced IR-induced decline by 23 %. However, long-term treatment with a clinical dose (10 mg/kg/day) had no impact on proliferation but significantly increased young neurons with radial dendrites (29 % in controls, 156 % after IR) and enhanced dendritic growth. Additionally, long-term memantine reduced NGF levels by 40 % without affecting BDNF [178].

### Anti-depressants

All classes of antidepressant drugs tested thus far, including 5-HT reuptake inhibitors (SSRIs), tianeptine, and mood stabilizers such as lithium (see below), were shown to increase the proliferation and survival of new neurons in the dentate gyrus [112,[179], [180], [181], [182], [183], [184], [185]]. Similarly, under chronic treatment conditions, CRHR1 and V1b receptor antagonists [183,186], improved deficits in neurogenesis caused by chronic mild stress [187]. Some of these compounds were tested for their effect on Alzheimer's disease. For example, Escitalopram Oxalate SSRI reduced Aβ production by increasing α-secretase cleavage of APP and reduced amyloid load [188]. Likewise, tianeptine reduced depressive symptoms and improved cognition in AD patients [189]. Fluoxetine decreased soluble Aβ_40_ and Aβ_42_ levels, attenuated astrocytic activation and reduced glial fibrillary acidic protein levels in APP/PS1 mice and alleviates APP phosphorylation [190].

Chronic administration of antidepressants such as fluoxetine, reboxetine, tranylcypromine, and electroconvulsive shock (ECS) enhances neurogenesis in adult rodents [179], with similar effects observed in non-human primates for fluoxetine and ECS [191]. Antidepressants targeting different neurotransmitter systems, including serotonin and norepinephrine, as well as SSRIs (citalopram, escitalopram), tricyclics (imipramine), mood stabilizers (lithium), and atypical antidepressants (AMPA receptor potentiators, agomelatine), promote neurogenesis and cell proliferation [181,192,193]. Transcranial magnetic stimulation, similar to ECS, also stimulates granule cell precursor proliferation [194]. However, the effects of anxiolytics on hippocampal neurogenesis remain inconclusive, though increased neurogenesis may help mitigate anxiety and depression in mice [195]. Endogenous GABAergic signaling has been proposed to promote granule cell maturation, as new granule neurons initially receive GABAergic inputs [196]. Physical activity, such as running, enhances this signaling and accelerates granule cell maturation in the dentate gyrus. Notably, the anxiolytic-like effects of running are blocked by GABA receptor antagonists in mice, suggesting a shared mechanism with anxiolytic medications [195,196].

**Valproic acid (VPA)** is used for the treatment of epilepsy and bipolar disorders, as well as the prevention of headaches and migraines. It acts on γ amino butyric acid (GABA) levels in the brain, blocks voltage-gated ion channels, and also acts as an HDAC inhibitor [197]. Evidence concerning the effect of VPA on neurogenesis is controversial, with some studies reporting that VPA enhanced hippocampal neurogenesis in the FAD-linked 3XTgAD mouse model via Wnt/β-catenin pathway [198]. Improved memory deficits and decreased Aβ deposition in AD mice was accompanied by attenuated neuroinflammation and neuronal degeneration [199]. Others report a suppressive effect on neurogenesis and cognitive function [200]. Cognitive impairments were observed in about 20 % of human patients that were treated with VPA [[201], [202], [203], [204], [205], [206], [207]]. Furthermore, valproic acid exhibits gender-specific effects on cognitive function and synaptic integrity in AD mice [208]. Similarly, valproic acid mitigated the gliotoxic effects of Aβ and decreased S100B levels, suggesting a potential neuroprotective role [209]. VPA and lithium have been reported to reduce tau protein phosphorylation both in vitro and in vivo by modulating GSK3 β.

**Levetiracetam (LEV)** is an anti-epileptiform activity compound that is thought to affect GABA turnover [210]. It was tested in FAD mouse models and in human patients with and without epileptic episodes. LEV improved cognitive function in FAD mice [211] and in AD patients with epileptiform activity [212].

**Lithium** treatment is associated with a decrease in cerebrospinal fluid concentrations of P-tau and improved cognitive performance in AD patients [213]. In transgenic mouse models, lithium stimulates neurogenesis and counteracts cognitive impairments by inhibiting GSK-3β and activating Wnt/β-catenin signaling pathways [214]. Additionally, short-term treatment with lithium effectively prevents the formation of NFTs in tau transgenic models [215]. The hippocampus is an important target of lithium, and was observed to enhance the differentiation of human hippocampal neural progenitor cells in culture [216].

### Metformin and anti-type-2-diabetes medications

Metformin, a widely used drug for the treatment of type 2 diabetes, is known to be an activator of adenosine monophosphate-activated protein kinase (AMPK) [217]. AMPK functions as an energy balance sensor and is rapidly activated in response to low energy supply [218]. AMPK is abundant in the brain, and its activation in the brain rapidly occurs in response to cerebral ischemia, which significantly increases angiogenesis and neurogenesis [219]. Chronic metformin treatment after stroke has been shown to improve functional recovery following ischemic stroke [[220], [221], [222]].

#### Herbal compounds that modulate neurogenesis

Herbal components have the potential to enhance hippocampal neurogenesis through diverse mechanisms (Table 3). For instance, various herbal components, such as curcumin, tanshinone I, and salvianolic acid B, exhibit the ability to enhance cognitive function and hippocampal neurogenesis by inhibiting GSK3. The involvement of GSK-3 in the progression of AD as a crucial molecular intermediary between senile plaques and neurofibrillary tangles is well-documented [223,224]. Studies have indicated that activated AKT hinders GSK-3β by phosphorylating it at serine 9. The PI3K/AKT/GSK-3β pathway plays a significant role in cell survival. Additionally, the PI3K/AKT pathway is essential for the survival, growth, proliferation, and migration of cells. In the context of AD, Aβ impedes the PI3K/AKT pathway, leading to neuronal degeneration and eventual dementia. Therefore, activation of the PI3K/AKT pathway is beneficial for inhibiting GSK-3β and preventing Aβ-induced toxicity. Analysis of data reveals that herbal compounds such as curcumin enhance cognitive function and hippocampal neurogenesis by modulating the PI3K/AKT signaling pathway under both in vitro and in vivo conditions. Numerous studies have highlighted the role of the PI3K/AKT signaling pathway activation in mediating the anti-AD effects of curcumin, puerarin, salvianolic acid B, and Ginseng components. It is now widely recognized that the pathogenesis of AD extends beyond proteinopathy and involves intricate interactions with the innate immune response in the brain. Aggregated proteins bind to pattern recognition receptors on glial cells, triggering an innate immune response characterized by the release of inflammatory cytokines, thereby impacting adult neurogenesis [225,226]. Recent research has affirmed the anti-inflammatory properties of several herbal components that enhance neurogenesis and alleviate AD progression [227]. For example, curcumin has been shown to mitigate neuroinflammatory responses by suppressing the NF-κB signaling pathway in an AD model. Notably, the suppression of neuroinflammation and enhancement of synaptic connectivity by curcumin contribute to hippocampal neurogenesis and cognitive performance in AD models. Similarly, evodiamine, a quinolone alkaloid derived from evodia rutaecarpa, has been demonstrated to enhance spatial learning and cognitive functions in APP/PS1 transgenic mice by inhibiting the inflammatory cascade [227]. In line with the pathological mechanisms of AD, the therapeutic benefits of herbal components can target various aspects of neurogenesis.

**Table 3:** Herbal drugs that modulate neurogenesis.

Herbal drug	Model	Mechanisms	Findings	Ref
*Curcuma longa* (Curcumin)	In vivo (β-amyloid treated rats) In vitro (culture of hippocampal NSCs)	↑ Wnt/β-Catenin Pathway ↓ GSK3beta levels	Increased hippocampal neurogenesis Enhanced learning and memory functions	(305)
	In vivo (APP/PS1 mice) In vitro (hippocampal neuronal/glial culture of rat embryos)	↓ NF-κB signaling pathway ↑ Activation of PPAR γ pathway	Improved spatial learning and memory Reduced neuroinflammatory response and β-amyloid-induced neuroinflammation	(306)
	Normal mice	↑ MAPK signaling	Enhanced hippocampal neurogenesis, synapse structure and function, Survival of newborn neurons, Proliferation of neural progenitor cells	(307)
	APP/PS1 mice	↓ PSD95 and Shank1 dysregulations	Improved synapse structure and function	(308)
	APP/PS1 mice	↑ PI3K/AKT signaling pathway	Enhanced learning and memory abilities	(309)
*Glycyrrhiza uralensis* (Liquiritin)	β-amyloid1-42- Treated rats	↑ Oxidative stress inhibition	Enhanced hippocampal neurogenesis, Reduced neuronal apoptosis, Mitigated memory and cognitive deficits	(310)
*Astragalus mongholicus* (Astragaloside)	In vivo (β-amyloid25–35 -treated mice) In vitro (cortical cultures)	↑ Activation of M1 muscarinic receptor	Enhanced spatial learning and memory, Reduced axonal atrophy and synaptic loss, Increased cytoskeletal molecules and cell survival	(311)
*Evodia rutaecarpa* (Evodiamine)	APP/PS1 mice	↑ Anti-inflammatory activity ↓ COX-2 protein	Improved spatial learning and memory	(312)
*Pueraria lobota* (Puerarin)	β-amyloid1-42- Treated rats	↓ Caspase-9 levels in hippocampus ↑ PI3K/Akt signaling	Enhanced spatial learning and memory, Reduced neuronal apoptosis in the hippocampus	(313)
	SAD cybrid cells	↓ Pro-death signaling pathways ↓ Caspase-3 activity ↑ Oxidative stress inhibition	Reduced neuronal apoptosis, Increased cell viability	(314)
*Glycyrrhiza glabra* (Glabridin)	Kunming mice	↑ Anti-inflammatory activity ↑ Oxidative stress inhibition ↑ AChEI	Improved spatial learning and memory	(315)
*Salvia miltiorrhizae* (Tanshinone I)	ICR mice	↑ GSK3beta inhibition ↑ Wnt/β-catenin signaling	Enhanced hippocampal neurogenesis, Increased granular neurons in hippocampus, Differentiation and proliferation of neuroblasts	(316)
*Scutellaria baicalensis* (Baicalein)	In vivo (Tg2576 mice) In vivo (CHO/APPwt Cells)	↑ GABAA receptor signaling	Improved cognitive performance, Increased nonamyloidogenic APP	(317)
*Panax ginseng* (Ginsenoside Rg1)	D-gal-induced aging rats	↑ Anti-inflammatory activity ↑ Oxidative stress inhibition	Enhanced hippocampal neurogenesis, Increased NSPCs survival and proliferation, Improved cognitive ability	(318)
*Panax ginseng* (Ginsenoside Rg1 and Rb1)	Normal mice	↑ AChEI	Improved spatial learning and cognitive functions Increased hippocampal synaptic density	(319)
*Panax ginseng* (Ginseng protein)	D-gal/AlCl3-Induced rats	↑ PI3K/AKT signaling pathway	Improved spatial learning and memory, Reduced β-amyloid contents, Enhanced neuroprotective effects	(320)
*Rehmannia glutinosa* (Catalpol)	In vivo (ICR mice) In vivo (culture of forebrain neurons)	↑ BDNF ↓ TrkB activity	Mitigated learning and memory deficits Increased cholinergic positive neurons Enhanced neurite outgrowth Increased muscarinic receptor density	(321)
*Salvia miltiorrhiza* (salvianolic acid B)	Culture of mice cortical NSCs	↑ GSK3beta inhibition ↑ PI3K/AKT signaling pathway	Enhanced NSCs differentiation and proliferation Increased neurite outgrowth	(322)
	Culture of rats cortical NSPCs; cerebral ischemia rats	↑ PI3K/AKT signaling pathway	Enhanced hippocampal neurogenesis, Increased granular neurons in hippocampus, and increased NSPCs differentiation and proliferation Improved learning and memory functions	(323)
*Epimedium brevicornum* (Icariin)	Tg2576 mice	↑ BDNF ↓ TrkB activity	Enhanced hippocampal neurogenesis Improved memory function Reduced cerebral β-amyloid and APP levels	(324)
	β-amyloid1-42- Treated rats	↑ BDNF ↓ TrkB	Improved spatial learning and memory activity Enhanced synaptic plasticity	(325)
*Herba Epimedii Maxim* (Icariside II)	APP/PS1 mice	↓ *p*-eIF2α signaling pathway ↓ p-PERK signaling pathway ↑PPARγ signaling pathway	Reduced β-amyloid production Improved spatial learning and memory Mitigated neuronal degradation	(326)
*Radix Polygalae* (Onjisaponin B)	APP/PS1 mice	↓ BACE-1 expression ↑ Anti-inflammatory activity	Enhanced amyloid precursor protein degradation, Reduced β-amyloid production	(327)
*Ginkgo Biloba* (Ginkgolide)	In vivo (APP/PS1 mice) In vitro (cortical cultures)	↑ CREB signaling	Enhanced hippocampal neurogenesis Increased hippocampal synaptogenesis NSCs differentiation and proliferation	(328)
*Ginkgo Biloba* (Bilobalide, quercetin and ginkgolide B)	Cultures of rat hippocampal neurons	↑ CREB signaling	Enhanced hippocampal neurogenesis Increased hippocampal synaptogenesis NSCs differentiation and proliferation	(329)
*Angelica sinensis* (Vanillic acid)	ICV-STZ mice	↑ Oxidative stress inhibition ↑ AChEI ↓ TNF-α activity ↓Corticosterone level	Improved spatial learning and memory Enhanced neuroprotective effects	(330)
*Polygala Tenuifolia* (Senegenin)	PC 12 ​cells	↑ Oxidative stress inhibition ↑ Inhibition of ASK1 and/or JNK pathways	Increased cell viability Enhanced neurite outgrowth Improved neural plasticity Reduced β-amyloid cytotoxicity	(331)
	In vivo (scopolamine-induced amnesia model of rats) In vitro (cultured neurons of cerebral cortex rat)	↑ AChEI	Improved spatial learning and memory Reduced toxicity in rat cortical neurons Mitigated neuronal degradation	(332)
*Camellia Sinensis* (Epicatechin)	Aging model mice	↑ CREB/ERK signaling	Increased total spine density Enhanced hippocampal neurogenesis Improved spatial learning and memory Increased newborn hippocampal cell survival	(333)

AD Alzheimer's disease, AChEI acetylcholinesterase inhibitor, NSCs neural stem cells, BDNF brain-derived neurotrophic factor, NPCs neural precursor cells, CREB cAMP response element-binding protein, GSK3beta glycogen synthase kinase 3 beta, MAPK Mitogen-activated protein kinase, APP/PS1 amyloid precursor protein/presenilin-1, PI3K/AKT phosphatidylinositol-3-kinase/protein kinase B, SAD sporadic Alzheimer's disease, ICV-STZ intracerebroventricular-streptozocin.

Firstly, they can enhance cell survival. For instance, puerarin derived from pueraria lobota has exhibited favorable effects on preventing neural apoptosis in SAD cybrid cells by inhibiting pro-death signaling pathways [227]. Moreover, puerarin has the potential to enhance spatial learning and cognitive functions by reducing neuronal apoptosis in a rat model treated with β-amyloid1-42 [228]. Additionally, liquiritin extracted from glycyrrhiza uralensis has shown promise in curbing neural apoptosis in the hippocampus of an AD rat model. Liquiritin significantly improves memory and cognitive deficits by mitigating oxidative stress [229].

(ii) Enhancement of cell proliferation and differentiation was observed, specifically through the use of asarones derived from rhizoma acori tatarinowii, resulting in increased hippocampal neurogenesis and recognition memory in a mouse model of Alzheimer's disease. Moreover, asarones were found to promote the proliferation and survival of neural stem cells when cultured in vitro [230]. Another compound, Tanshinone I, extracted from salvia miltiorrhizae, also exhibited enhancement in cell proliferation and differentiation in the DG in mice [231]. The use of Salvia miltiorrhiza, particularly salvianolic acid B, demonstrated improvements in learning and memory functions as a potential therapeutic approach for neurodegenerative disorders. Additionally, salvianolic acid B was shown to enhance differentiation and proliferation of neural stem/progenitor cells, as well as hippocampal neurogenesis both in vivo and in vitro [232,233]. The action of Panax ginseng, specifically ginsenoside Rg1, was found to play a role in promoting the survival and differentiation of neural stem/progenitor cells, ultimately contributing to neurogenesis. Furthermore, ginsenoside Rg1 exhibited a preventative effect against cognitive decline in an aging rat model of neurodegeneration [234].
(iii) Facilitation of synaptic connections was achieved through the use of astragaloside, extracted from astragalus mongholicus, which led to improvements in memory impairment and significant regeneration of synapses and neurites in the hippocampus and cerebral cortex of mice with Alzheimer's disease. Various astragalosides isolated from astragalus mongholicus extract were also found to enhance axonal outgrowth, provide neuroprotective effects, and increase cell viability in the hippocampus and cerebral cortex of mice [235]. The administration of Rehmannia glutinosa, specifically catalpol, resulted in improved memory and cognitive function in mice with neurodegeneration. Catalpol was reported to have a protective role in increasing neuron numbers, muscarinic receptor density, and length of neurite outgrowth in cultured forebrain neurons [236]. Treatment with Epimedium brevicornum, containing icariin, showed enhancement of memory function, reduction in β-amyloid peptide content, decreased expression of amyloid precursor protein in the brain, and promotion of hippocampal neurogenesis and synaptic plasticity in mouse [237,238] and rat [239] models of AD. Ginkgo biloba, specifically ginkgolide, when orally administered to a mouse model of AD, showed improved learning and memory with beneficial effects on the hippocampus [240]. Additionally, ginkgo biloba leaf extract containing quercetin and bilobalide was found to enhance neurogenesis and synaptogenesis in hippocampal neural stem cells when cultured in vitro [241]. Panax ginseng, with its components ginsenoside Rg1 and Rb1, was shown to enhance spatial learning and cognitive functions in young mice, while also increasing hippocampal synaptic density. Selective enhancement of cognitive function and hippocampal neurogenesis are considered desirable traits of an effective treatment of AD [242].

#### Physical activity impact on adult neurogenesis

An intriguing association exists between physical exercise and adult hippocampal neurogenesis, as physical activity is known to enhance neurogenesis in the SGZ and the SVZ [243,244]. Early studies by Van Praag et al. in mice, revealed that voluntary exercise on a running wheel doubled cell proliferation and neurogenesis in the DG [245]. Conversely, a separate study found that swimming exercise increased progenitor cell proliferation and maturation in the SVZ in rats [246].This increase was attributed to greater levels of the trophic factor NGF, potentially aiding in neurogenesis post-activity [246]. The effect on neurogenesis may vary based on type of physical activity, endurance, duration and frequency, species-specificity to name a few variables. Kronenberg et al. indicated that sustained running in mice led to a temporary spike in NSC proliferation, alongside a notable increase in doublecortin-positive immature neurons despite a return to baseline NSC proliferation levels in the DG [243]. This spike in DG neurogenesis post-exercise was associated with enhanced spatial memory, implying that consistent exercise could enhance cognitive function through increased neurogenesis [247]. Additionally, physical activity could maintain neuronal plasticity and enhance learning, as evidenced by improved performance in water maze tests in mice following wheel running [244]. Notably, the inhibition of neurogenesis through focal irradiation eliminated exercise-induced improvements in spatial learning, supporting the connection between exercise, hippocampal neurogenesis, and cognitive enhancement [248].

Physical activity enhances hippocampal neurogenesis and cognitive function by boosting cerebral blood flow [249], BBB permeability [250], angiogenesis [251], and the expression of neurotrophic factors [252]. Here, we delve into the role of neurotrophic factors as mediators of the effects of physical activity on inducing neurogenesis. Studies have shown that physical activity elevates levels of neurotrophic factors like NGF [246], IGF-1 [253], VEGF [254], and BDNF [255] potentially explaining how exercise enhances adult neurogenesis.

For example, Lafenêtre et al. engineered mice with reduced hippocampal cell proliferation and short-term memory through genetic modifications. Following running activity, these mice displayed a reversal of the genetic blockage in cell proliferation and an improvement in short-term memory. Running also led to an increase in BrdU- and DCX-positive cells in the hippocampus of these mice. Notably, an elevation in the BDNF receptor TrKB was observed in DCX-positive cells post-running, hinting at a potential signal for increased hippocampal neurogenesis [256]. The suppression of IGF-1 signaling through the use of a particular antibody that targets the IGF-1 receptor led to the impairment of exercise-induced cognitive function in rats [257]. Moreover, reports have indicated that aerobic exercise promoted the uptake of IGF-1 by specific neurons in the rat brain, resulting in the spontaneous activation of neurons and an increase in the expression of BDNF [258]. The combination of BDNF and exercise reversed AD pathology in mice [259]. Alongside BDNF and IGF-1, VEGF has been demonstrated to possess neurotrophic properties, with elevated levels being observed post-exercise in rats [260]. Blocking VEGF in the periphery hindered the enhanced neurogenesis triggered by running, while such blockade had no impact on the baseline neurogenesis in sedentary mice [261], underscoring the role of VEGF in exercise-induced adult neurogenesis. A clinical analysis exploring high and moderate intensity exercise groups unveiled a rise in BDNF and IGF-1 concentrations, correlating with improved cognitive performance compared to low-intensity exercise groups [262]. These investigations emphasize the heightened secretion of growth factors as the basis for the favorable neurophysiological outcomes of physical exercise. Consequently, regular physical activity may serve as a non-invasive method to stimulate the intrinsic expression of neurotrophic factors, thereby promoting neurogenesis.

#### Caloric restriction and intermittent fasting

Various preclinical models have documented an enhancement in AHN under conditions of intermittent fasting (IF) or caloric restriction (CR). CR models typically involve a reduction of 30–40 % in daily caloric consumption, while animal IF models encompass *ad libitum* feeding with periods of 8 h/16 h or 12 h/12 h without caloric restriction.

There exists a widespread agreement that both IF and CR foster AHN. This is evidenced by an increase in the population of immature neuroblasts [263], enhancement in the survival of neuronal precursor cells leading to their maturation into fully developed neurons [264], proliferation of shuttle-shaped cells in the SGZ, augmentation of cell density in CA3, and elevation in the quantity of neurons and glial cells [265]. Only a singular study has indicated a reduction in new hippocampal neuron formation in response to IF [266].

Numerous researchers have put forth varied mechanisms to explain the induction of AHN by IF and CR [267]. Both dietary paradigms have been linked to an overall upsurge in the expression of neurotrophic factors, such as neurotrophin-3 [268], ciliary neurotrophic factor [269] and BDNF [270], notably within the newly generated neurons of the DG [267]. Animals subjected to CR exhibited heightened expression of genes associated with neuronal safeguarding and differentiation, such as NeuroD1 [271], Notch [264], Klotho [263], Egr1 [272].

Moreover, dietary restriction has been associated with alterations in epigenetic regulation. Animals undergoing CR displayed a mitigation of age-related CG/CH methylation and a prevention of age-related hypermethylation [273], while IF stimulated the inhibition of histone deacetylase [274] and concurrently promoted the deacetylation of genes that delay cellular aging processes [275]. Additionally, CR was linked to a reduction in the expression of versican, a protein implicated in age-related functions crucial for neuronal development, maturation, and survival [266]. Animals adhering to a restricted diet exhibited an elevation in microRNA MMV-MIR-713, with CR enhancing gene ontology related to the prediction of microRNA targets involved in neuronal generation, differentiation, and development [276]. CR also increased the expression of the PARP gene, which is connected to DNA repair mechanisms and chromatin remodeling [266].

Several studies have emphasized the correlation between neurogenesis induced by IF and CR and the upregulation of hippocampal NPY levels [277], as well as the reliance on increased levels of hippocampal acyl-ghrelin and ghrelin receptor [272]. Noteworthy is the fact that IF and CR also triggered a CREB-mediated elevation in SIRT1 and SIRT3 [275]. Limited evidence has also suggested a role for GSK3β [271].

The activation of the Foxo3 gene by CR leads to enhanced adult neurogenesis by reducing neuroinflammation [278]. In this context, there was a decrease in proinflammatory hormones and cytokines [274], diminished glial activation [271], and increased levels of IFN-γ (which promotes neural differentiation and neurite outgrowth in neural adult stem cells) [279].

Ultimately, IF and CR resulted in a decrease in hippocampal oxidative stress and total protein oxidation levels by enhancing catalase reactivity and superoxide dismutase activity [280], in conjunction with the upregulation of heat shock protein 70 and glucose-related protein 78 expression (both involved in protecting against oxidative stress) [275].

#### Environmental enrichment (EE)

Various animal studies have underscored the positive effects of an EE on hippocampal structures. Exposure to EE has been shown to enhance hippocampal neurogenesis [281]. EE has been found to stimulate the proliferation of progenitor cells and enhance cell survival in the hippocampus [282]. This phenomenon has also been observed in transgenic rodent models of AD, where EE reinstated impaired adult hippocampal neurogenesis post Aβ plaque deposition [283].

Furthermore, both the volumes of CA1 and DG in the hippocampus exhibited significant increments following prolonged exposure to EE, leading to enhanced cognitive performance [284]. The rise in hippocampal volume could be linked to the cumulative impacts of EE, including increased cell proliferation, dendritic arborization, improved vascularity, and enhanced dendritic complexity [285]. The effects of EE on hippocampal activity have also garnered attention in recent times. Hippocampal activity is intricately connected to the long-term potentiation (LTP) generated between hippocampal excitatory neurons, crucial for learning and memory formation processes [286]. Additionally, synaptic dysfunction in the hippocampus is implicated in the early stages of AD, contributing to the gradual loss of memories [287]. Recent studies on AD utilizing diverse transgenic rodent models have demonstrated a notable decline in the magnitude of hippocampal LTP through electrophysiological recordings, indicating reduced synaptic plasticity in the hippocampus [288]. Synaptic loss and dysfunction have been strongly associated with cognitive decline in individuals with AD [289].

The impact of EE on synaptic plasticity within the hippocampus has been a topic of frequent discussion in various rodent models. Enhanced hippocampal long-term potentiation (LTP) has been observed following exposure to short-term EE [290]. Analysis of deep sequencing across the whole brain post-EE exposure demonstrated an increase in the expression of genes related to synaptic plasticity, including BDNF and the N-methyl d-Aspartate receptor subtype 2B (GRIN2B) genes [284]. In rodent models prone to seizures, EE was able to maintain hippocampal LTP in CA1 neurons, while also preventing the loss of synaptic density and dendrite branching [291]. Recent research delved into the impact of EE on hippocampal plasticity in mice models with permanent middle cerebral artery occlusion. Following 28 days of EE exposure, there was a significant elevation in the expression of synaptic proteins such as growth-associated protein 43 (GAP-43), synaptophysin, and postsynaptic density protein 95 (PSD-95) compared to mice in standard housing, resulting in increased hippocampal synapse formation [292]. Additionally, similar effects were observed in transgenic AD rodents, particularly in enhancing synaptic plasticity in transgenic AD mice carrying mutations in the APP and PS1 genes (APPswe/PS1ΔE9). Furthermore, a 4-week exposure to EE prevented synaptic dysfunction induced by Aβ Oligomer deposition [293], highlighting the potential of EE as a strategy for both neuroprotection and treatment.

## Future Remarks

In summary, the regulation of neurogenesis exhibits great potential as a therapeutic approach to augment hippocampus plasticity and ameliorate cognitive performance in both aging and Alzheimer's disease. This complex process is orchestrated by several regulatory mechanisms and is influenced by both intrinsic and extrinsic factors. Comprehending these mechanisms provides a foundation for developing interventions that can stimulate the production and integration of new neurons, which may help to reverse or lessen the cognitive impairments associated with aging and neurodegenerative diseases.

To further this therapeutic potential, future studies should concentrate on the following important areas:1. Understanding Molecular and Cellular Mechanisms: It is imperative to better understand the molecular and cellular mechanisms regulating adult hippocampus neurogenesis. Identifying molecular targets within these pathways can facilitate the development of more precise and effective therapeutic agents.2. Translation to Human Models: Despite great advancements in animal models, a crucial obstacle still exists in applying these discoveries to human biology. By using cutting-edge techniques to study neurogenesis in humans, such as the use of human-derived neural stem cells and sophisticated imaging technologies to study neurogenesis in humans., research should try to close this gap.3. Combination Therapies: Researching the synergistic effects of combining medication, cognitive training, physical exercise, and other therapeutic modalities with neurogenic stimulators may improve overall efficacy and offer a more all-encompassing approach to halting cognitive decline.4. Long-term Efficacy and Safety: Researching neurogenic treatments' long-term impacts and safety is crucial to their clinical use. This involves comprehension. Possible adverse effects, the best dosage schedules, and the long-term sustainability of therapeutic advantages.5. Personalized Medicine: Individual differences in neurogenic capacity and response to therapy may enable tailored therapeutic strategies. Customizing therapies according to environmental, genetic, and epigenetic factors may enhance results and lower the possibility of negative side effects.

Through tackling these domains, forthcoming investigations may facilitate novel therapies that leverage neurogenesis's capacity to augment hippocampus plasticity and cognitive performance, ultimately elevating the standard of living for those impacted by aging and Alzheimer's disease.

## Author contribution

MM reviewed the literature and wrote the review, AD generated data and wrote the review, OL overviewed research content and manuscript writing.

## Declaration of competing interest

The authors declare the following financial interests/personal relationships which may be considered as potential competing interests: Orly Lazarov reports financial support was provided by National Institute on Aging. N/A If there are other authors, they declare that they have no known competing financial interests or personal relationships that could have appeared to influence the work reported in this paper.
